# Early Ocular-Surface Changes During Dupilumab Treatment According to Hyaluronic Acid Use: A Prospective Cohort Study

**DOI:** 10.3390/jpm16090476

**Published:** 2026-09-16

**Authors:** Giuseppe Demichele, Giulia Ciccarese, Aurora De Marco, Giovanni Petruzzella, Chiara Barlusconi, Cristiana Mileti, Giovanni Alessio, Maria Gabriella La Tegola, Rosa Anna Favale, Francesca Ambrogio, Alexandre Raphael Meduri, William Andrew Rosato, Rossana Spadavecchia, Paolo Romita, Domenico Bonamonte, Caterina Foti

**Affiliations:** 1 Section of Dermatology and Venereology, Department of Precision and Regenerative Medicine and Ionian Area (DiMePRe-J), University of Bari “Aldo Moro”, 70124 Bari, Italy; giuseppe.demichele08@gmail.com (G.D.); aurorademarco94@gmail.com (A.D.M.); francesca.ambrogio@policlinico.ba.it (F.A.); a.meduri@phd.uniba.it (A.R.M.); william.rosato@uniba.it (W.A.R.); rossana.spadavecchia@policlinico.ba.it (R.S.); paolo.romita@uniba.it (P.R.); domenico.bonamonte@uniba.it (D.B.); caterina.foti@uniba.it (C.F.); 2 Section of Ophthalmology, Department of Translational Biomedicine and Neuroscience (DiBraiN), University of Bari “Aldo Moro”, 70124 Bari, Italy; giovanni.petruzzella@policlinico.ba.it (G.P.); giovanni.alessio@uniba.it (G.A.); mariagabriella.lategola@uniba.it (M.G.L.T.); rosaanna.favale@policlinico.ba.it (R.A.F.); 3 Dermatology Unit, Ospedale di Circolo, ASST Sette Laghi, 21100 Varese, Italy; chiara.barlusconi@asst-settelaghi.it; 4 Section of Radiology and Radiation Oncology, Interdisciplinary Department of Medicine, University of Bari “Aldo Moro”, 70124 Bari, Italy; cristiana.mileti@policlinico.ba.it

**Keywords:** atopic dermatitis, dupilumab, dupilumab-associated ocular surface disease, dry-eye disease, hyaluronic acid, Schirmer test, tear production, ocular surface

## Abstract

**Background**: Ocular-surface abnormalities are common in patients with moderate-to-severe atopic dermatitis and may predispose to dupilumab-associated ocular surface disease (DAOSD). We investigated early ocular-surface changes during dupilumab treatment and whether baseline-guided hyaluronic acid (HA) eye-drop use was associated with more favorable ocular outcomes. **Methods**: In this prospective single-center cohort study, patients with moderate-to-severe atopic dermatitis underwent ophthalmologic assessment before dupilumab initiation and after 4 months. HA 0.15% eye drops were prescribed to patients with at least one basal or reflex Schirmer value ≤15 mm and ocular symptoms. Ocular outcomes included tear break-up time (TBUT), basal and reflex Schirmer tests, meibography, and the Ocular Surface Disease Index (OSDI). Follow-up values were compared between HA users and non-users using baseline-adjusted models with false-discovery-rate correction. **Results**: Thirty-nine patients were included; 18 received HA eye drops and 21 did not. Twenty-seven patients (69.2%) had at least one baseline Schirmer value ≤15 mm. At 4 months, reflex Schirmer values were higher in HA users than in non-users after baseline adjustment (adjusted mean difference, 5.60 mm; 95% CI, 1.64–9.57; *p* = 0.007; *q* = 0.035). In the low-Schirmer subgroup, HA use was associated with higher reflex Schirmer (8.50 mm; 95% CI, 4.84–12.17; *q* < 0.001) and basal Schirmer values (5.07 mm; 95% CI, 1.41–8.73; *q* = 0.022). Among untreated patients with low baseline Schirmer values, reflex tear production decreased in eight of nine patients, despite OSDI scores remaining within the conventional normal range. **Conclusions**: HA supplementation at dupilumab initiation was associated with more favorable changes in tear production, particularly among patients with reduced baseline Schirmer measurements. Objective deterioration in tear production despite persistently low OSDI scores highlights the limitations of symptom-based assessment alone. These findings support baseline ophthalmologic evaluation and combined objective and symptom monitoring during dupilumab treatment. Larger controlled studies are needed to determine whether early, targeted HA eye-drop use can prevent clinically relevant dupilumab-associated ocular surface disease.

## 1. Introduction

Atopic dermatitis (AD) is a chronic inflammatory skin disease characterized by pruritus, recurrent eczematous lesions, and a substantial impact on quality of life [1,2].

The diagnosis is made clinically and is based on historical features, morphology and distribution of skin lesions, and associated clinical signs such as atypical vascular responses (facial pallor, white dermographism), keratosis pilaris, pityriasis alba, ocular/periorbital changes, perifollicular accentuation, lichenification and others. Pruritus is a hallmark feature of the condition, accounting for a significant portion of the disease burden experienced by patients and their families [3].

Dupilumab, a monoclonal antibody that inhibits interleukin (IL)-4 and IL-13 signaling through blockade of the IL-4 receptor α, has demonstrated marked efficacy in moderate-to-severe AD, improving clinical signs, pruritus, and patient-reported quality of life [4]. However, conjunctivitis occurred more frequently in dupilumab-treated patients than in placebo recipients in AD trials and was associated with greater baseline AD severity and a previous history of conjunctivitis [4,5]. Prospective ophthalmologic studies have subsequently shown that the ocular spectrum extends beyond conjunctivitis to include dry-eye disease, blepharitis, and superficial punctate keratitis, supporting the broader concept of dupilumab-associated ocular surface disease (DAOSD) [6].

Importantly, ocular-surface abnormalities may already be present before dupilumab initiation and may remain underrecognized when assessment relies predominantly on symptoms. Prospective studies have documented a high burden of pre-existing ocular surface disease in patients with moderate-to-severe AD, with objective abnormalities not always accompanied by corresponding symptoms [7,8,9].

Baseline ocular impairment may also identify patients at greater risk of DAOSD during treatment: dry-eye disease at baseline was independently associated with dupilumab-induced blepharoconjunctivitis in a prospective multicenter study [10]. In addition, reductions in tear break-up time (TBUT) and Schirmer tests, procedures measuring tear-film stability and tear production, have been reported during dupilumab therapy [11], and low baseline TBUT has been identified as a predictor of subsequent ocular adverse events [12]. These findings support the concept that DAOSD may result, at least in part, from the interaction between pre-existing ocular-surface vulnerability and treatment-related changes.

The optimal preventive strategy for DAOSD, however, remains uncertain. Current expert consensus considers the evidence insufficient to support routine prophylactic lubricant eye-drop use in patients without pre-existing ocular disease, while emphasizing appropriate management of patients with established ocular-surface abnormalities [13]. Hyaluronic acid (HA) is widely used in dry-eye disease and has a favorable safety profile, with evidence of improvement in ocular-surface signs and symptoms [14]. In dupilumab-treated patients, individualized ophthalmologic management including artificial tears has been associated with a lower observed incidence of conjunctivitis in a nonrandomized study [15]. Moreover, an open-label prospective study reported a lower incidence of dupilumab-associated conjunctivitis with prophylactic diquafosol, an aqueous secretagogue that stimulates P2Y2 receptors in the cornea and conjunctiva, promoting water secretion and enhancing mucin release from conjunctival goblet cells [16].

However, whether a baseline-guided strategy using HA is associated with preservation of normal quantitative ocular-surface parameters remains unclear [13].

We conducted a prospective cohort study to evaluate changes in ocular-surface parameters from baseline to 4 months post-dupilumab initiation between HA users (prescribed based on baseline clinical features and symptoms) and non-users. We aimed to examine whether HA use was associated with changes in ocular-surface parameters during dupilumab treatment.

## 2. Materials and Methods

### 2.1. Study Design and Setting

This was a single-center prospective cohort study involving patients with moderate-to-severe AD who initiated dupilumab at Policlinico di Bari, Italy. Ocular surface status was assessed at baseline, before dupilumab initiation (T0), and after 4 months of treatment (T1). Consecutive patients with moderate-to-severe AD initiating dupilumab during the study period were screened for eligibility and enrolled according to the predefined inclusion and exclusion criteria.

The study assessed early changes in ocular-surface parameters comparing patients prescribed HA eye drops at dupilumab initiation with non-users. HA use was nonrandomized and guided by predefined baseline ocular findings and symptoms. The resulting comparisons describe associations and do not establish causal effects of HA.

Participants initiated dupilumab between February 2021 and February 2022.

### 2.2. Inclusion and Exclusion Criteria

Patients were eligible for inclusion if they were aged 12 years or older, had dermatologist-confirmed moderate-to-severe AD, and were clinically eligible to initiate dupilumab because of inadequate disease control, intolerance, or contraindications to conventional systemic treatment. AD was diagnosed clinically by a dermatologist based on pruritus, typical eczematous lesions with age-appropriate morphology and distribution, and a chronic or relapsing course [3]. Severe disease was defined by an EASI score > 21. Exclusion criteria were ocular conditions requiring treatment that could interfere with ocular-surface assessment and concomitant systemic or topical ophthalmic treatments expected to materially affect ocular-surface measurements.

### 2.3. Ethics Approval

The study protocol (“Valutazione oculistica nel paziente affetto da dermatite atopica da moderata a grave eleggibile a trattamento con dupilumab: fattori di rischio e patogenesi”; study no. 6738) was approved by the Independent Ethics Committee of the Azienda Ospedaliero-Universitaria “Consorziale Policlinico”, Bari, Italy, on 24 February 2021.

### 2.4. Dupilumab Regimen and Hyaluronic Acid Eye Drops Allocation

All participants received dupilumab according to the standard regimen for AD, consisting of a 600 mg loading dose subcutaneously followed by 300 mg every 2 weeks.

HA eye drops were not prescribed universally. At baseline, their use was determined by a clinical rule requiring both:

1. At least one basal or reflex Schirmer measurement of 15 mm or less in either eye;
2. At least one ocular symptom elicited during direct ophthalmologic interview (ocular itching, burning, and/or dryness sensation).

The lubricant formulation, dosing frequency, and treatment duration were the following: 0.15% hyaluronic acid eye drops, one drop in each eye four times daily for 4 months. The use of HA-containing lubricants is supported by studies in dry-eye disease [14,17]. Continuation was verified, and all 18 HA patients maintained treatment without interruption throughout follow-up.

Patients in the non-HA group were instructed not to initiate HA or other topical ocular treatments expected to affect ocular-surface measurements during follow-up unless clinically indicated; any rescue treatment was to be recorded. No patient in the non-HA group required rescue therapy or initiated HA during the 4-month follow-up.

### 2.5. Ophthalmologic Assessment

A standardized ophthalmologic assessment was performed at baseline, before dupilumab initiation (T0), and after 4 months of treatment (T1). At baseline, previous ophthalmologic history, history of allergic conjunctivitis, periocular eczema, and current ocular symptoms were recorded separately. Current ocular symptoms included itching, burning, and/or dryness and were elicited during the baseline ophthalmologic interview.

At each visit, ocular-surface evaluation included BUT, reflex Schirmer testing, basal Schirmer testing, infrared meibography, and the 12-item Ocular Surface Disease Index (OSDI).

BUT is a standard test for tear-film stability performed by using standard fluorescein strips or minimal fluorescein solutions. Shorter BUT values indicate reduced tear-film stability. Values below 10 s are commonly considered abnormal, with values below 5 s indicating more marked tear-film instability [18].

The Schirmer test measures tear production to diagnose dry eye syndrome using 5 × 30 mm filter paper strips placed for 5 min between the palpebral conjunctiva and the eye’s bulbar conjunctiva. Without anesthesia, it measures combined basal and reflex (triggered by eye irritation from the filter paper) tearing, whereas topical anesthesia isolates basal secretion by preventing reflex tearing. Results are recorded as the length of the moistened paper. A score greater than 15 mm in 5 min is considered normal. A score of less than 15 mm in 5 min indicates the presence of dry eye [19].

Infrared meibography uses infrared wavelength light to visualize meibomian glands in vivo. These glands secrete the lipid meibum into the pre-corneal tear film to prevent tear evaporation and desiccation of the ocular surface. Meibomian gland dysfunction, which involves terminal duct obstruction, inflammation, keratinization, and eventual gland loss, is the main cause of evaporative dry-eye disease [20]. Meibography grading quantifies meibomian gland loss or atrophy using infrared imaging. Clinicians typically employ a 0–4 scale to evaluate the extent of glandular dropout in the upper and lower eyelids. Grade 0 indicates normal glands while grade 4 means advanced glandular atrophy [21].

The OSDI consists of a 12-item questionnaire designed to assess the visual disability caused by dry eye syndrome. The OSDI score can range from 0 to 100, with higher scores indicating greater disability; scores below 13 were considered within the conventional normal range [22].

BUT, Schirmer and meibography measurements were recorded separately for the right and left eyes.

A low-Schirmer subgroup was defined by the presence of at least one baseline basal or reflex Schirmer measurement of 15 mm or less in either eye regardless of the presence of symptoms.

### 2.6. Dermatologic Assessment

At T0 and T1, AD severity was assessed using the Eczema Area and Severity Index (EASI) and the Scoring Atopic Dermatitis (SCORAD) index. Patient-reported disease burden was evaluated using the Dermatology Life Quality Index (DLQI) and a numerical rating scale (NRS) for pruritus. Higher scores indicated greater disease severity or patient-reported burden [3]. The SCORAD sleep component (0–10) was also evaluated separately at T0 and T1. Baseline atopic comorbidities, non-allergic comorbidities, and previous AD treatments were also recorded.

### 2.7. Study Outcomes

The primary outcome was to evaluate 4-month changes from baseline in BUT, basal and reflex Schirmer test values, meibography grades, OSDI scores, and dry eye clinical symptoms in patients receiving HA eye drops compared to controls.

Additional study outcomes were to compare the dermatologic response to dupilumab between groups and to investigate the relationship between baseline dermatologic severity and baseline ocular-surface parameters.

### 2.8. Statistical Analysis

The study cohort comprised participants with documented lubricant eye drop allocation and complete T0 and T1 ocular assessments. For bilateral ocular measurements—BUT, reflex Schirmer, basal Schirmer, and meibography—the arithmetic mean of the right- and left-eye values was calculated for each participant at each ophthalmologic visit.

Continuous variables were summarized as mean and standard deviation, whereas categorical variables were reported as absolute values and percentages. Baseline differences between the HA and non-HA groups were analyzed using Welch’s *t* test for continuous variables and Fisher’s exact test for categorical variables. Welch’s *t* test does not require equality of variances between groups. Independence of observations followed from the study design, and continuous-variable distributions were assessed for marked departures from normality and extreme outliers before parametric comparisons; no major violations affecting the applicability of the test were identified.

For each ocular outcome, the primary between-group comparison was performed using linear analysis of covariance, with the T1 value as the dependent variable, HA group as the group indicator, and the corresponding T0 value as a covariate. Statistical inference, including 95% confidence intervals and *p* values, was based on heteroskedasticity-consistent type 3 (HC3) standard errors. Results were reported as adjusted mean differences between the hyaluronic acid and no-lubricant groups. The Benjamini–Hochberg procedure was applied across the five ocular outcomes to control the false discovery rate.

A subgroup analysis was conducted among participants with at least one baseline basal or reflex Schirmer measurement of 15 mm or less in either eye. Between-group comparisons within this subgroup used the same baseline-adjusted models applied in the complete cohort, with false-discovery-rate correction across the five ocular outcomes. Among low-Schirmer participants who did not receive lubricant, T0-to-T1 changes were additionally examined using paired Wilcoxon signed-rank tests. OSDI values below 13 were considered within the conventional normal range. These subgroup analyses were considered exploratory.

Dermatologic response was described using changes in EASI, SCORAD, DLQI, and pruritus NRS from T0 to T1. Between-group comparisons were based on linear models of the T1 value adjusted for the corresponding baseline value, with HC3 robust standard errors and Benjamini–Hochberg correction across the four outcomes.

All statistical tests were two-sided. *p* Values below 0.05 were considered nominally significant; for analyses subject to multiplicity correction, *q* values below 0.05 were considered significant after false-discovery-rate control. Analyses were performed using R version 4.6.1 (R Foundation for Statistical Computing, Vienna, Austria).

## 3. Results

### 3.1. Study Cohort and Baseline Characteristics

Of the 40 participants in the source cohort, 39 completed T0 and T1 dermatological and ocular assessments and were included in the analysis. One participant was lost to follow-up after ceasing to attend the scheduled follow-up visits.

Twenty-seven participants were assigned to the low-Schirmer subgroup (69.2% of the total) according to a baseline basal or reflex Schirmer test result of ≤15 mm. Among them, 18 symptomatic patients (46.1% of the global cohort) received HA eye drops (HA group), whereas the remaining nine asymptomatic patients received no HA treatment (non-HA group). An additional 12 participants with baseline Schirmer values above 15 mm and without symptoms were also assigned to the non-HA group.

**Figure 1:**
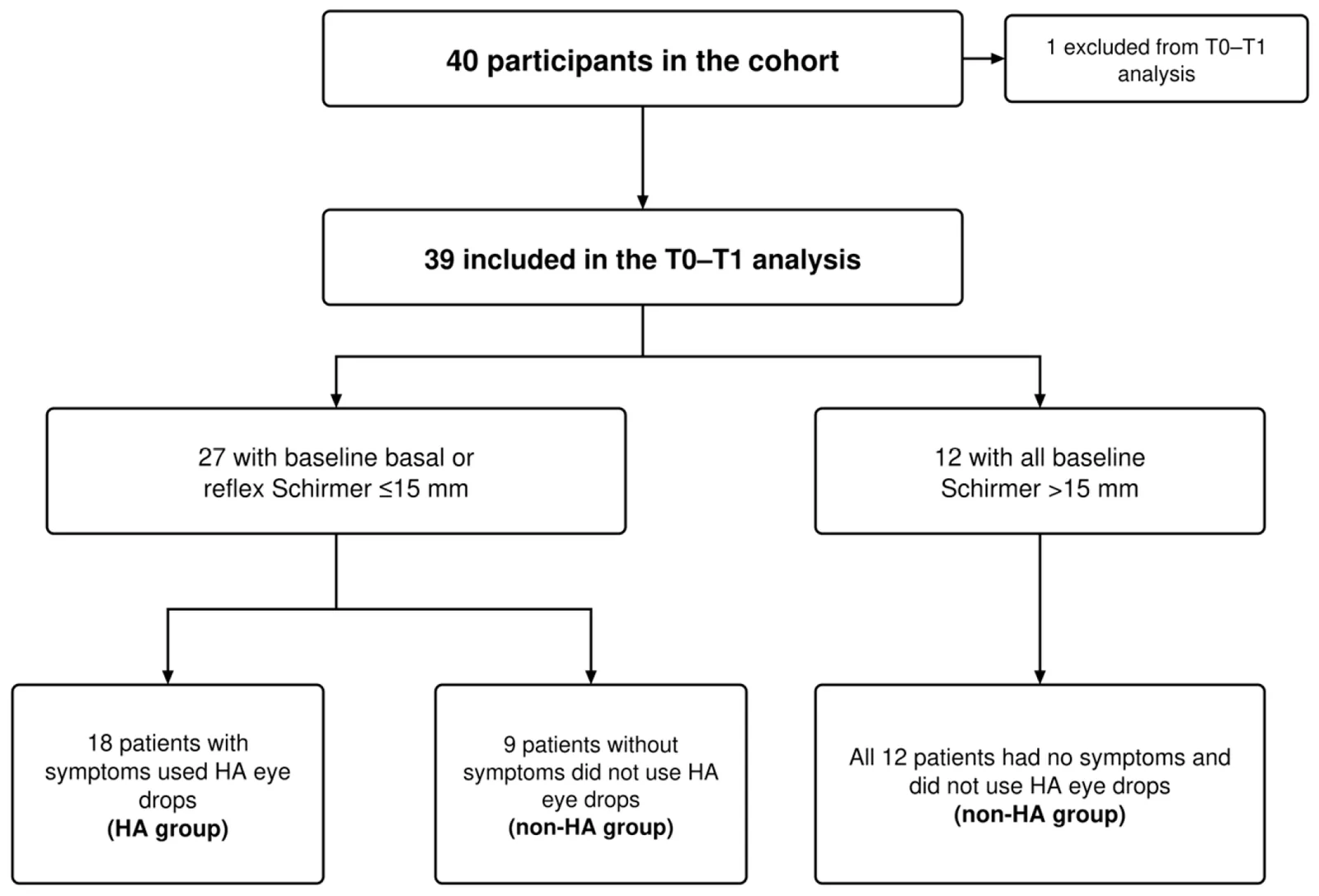
Study flow from cohort enrollment to the T0–T1 analysis, with allocation according to baseline Schirmer values, ocular symptoms, and HA eye-drop use. HA, hyaluronic acid; T0, baseline; T1, 4-month follow-up.

Overall, 18 participants received HA eye drops and 21 patients did not (Figure 1).

Baseline demographic characteristics were similar between groups, including age, and sex (Table 1). The mean age of the overall study cohort at dupilumab initiation was 40.95 ± 19.79 years. Baseline TBUT and meibography grades were also comparable. In contrast, participants of the HA group had lower reflex Schirmer values (10.31 vs. 18.14 mm), lower basal Schirmer values (12.92 vs. 19.81 mm), and higher OSDI scores (11.03 vs. 2.31) compared to the non-HA group. SCORAD was also higher in the HA group (73.63 vs. 65.03), whereas EASI was similar between groups. Atopic and non-allergic comorbidities were present in both groups. Allergic conjunctivitis was reported in 12/18 (66.7%) HA patients and 9/21 (42.9%) non-HA patients, allergic rhinitis in 16/18 (88.9%) and 13/21 (61.9%), allergic asthma in 7/18 (38.9%) and 6/21 (28.6%), and non-allergic comorbidities in 11/18 (61.1%) and 11/21 (52.4%), respectively. Previous ophthalmologic history was reported in 11/18 (61.1%) HA patients and 11/21 (52.4%) non-HA patients, while periocular eczema was present in 3/18 (16.7%) and 4/21 (19.0%), respectively. Previous AD treatment exposure was also broadly shared between groups: antihistamine, phototherapy, and topical calcineurin-inhibitor use were similar, while all patients had previously received topical corticosteroids. Previous systemic corticosteroid treatment was more frequent in the HA group (13/18, 72.2% vs. 10/21, 47.6%), whereas cyclosporine use was more frequent in the non-HA group (10/18, 55.6% vs. 15/21, 71.4%).

No other clinically relevant adverse events related to dupilumab were recorded during the 4-month follow-up.

### 3.2. Ocular Outcomes at 4 Months

Over the 4-month follow-up, mean TBUT decreased by 2.65 s in the non-HA group and increased by 1.73 s in the HA group. In the baseline-adjusted analysis, TBUT was 3.81 s higher in the HA group at T1 (95% CI, 0.39 to 7.24; *p* = 0.030), although this association did not remain significant after false-discovery-rate correction (*q* = 0.076).

Mean reflex Schirmer decreased by 4.40 mm without HA eye drops and increased by 4.64 mm with HA eye drops. The adjusted between-group difference was 5.60 mm (95% CI, 1.64 to 9.57; *p* = 0.007; *q* = 0.035). Basal Schirmer also showed more favorable changes over time in the HA group, but the adjusted difference was inconclusive (3.12 mm; 95% CI, −1.00 to 7.24; *p* = 0.133; *q* = 0.222).

**Figure 2:**
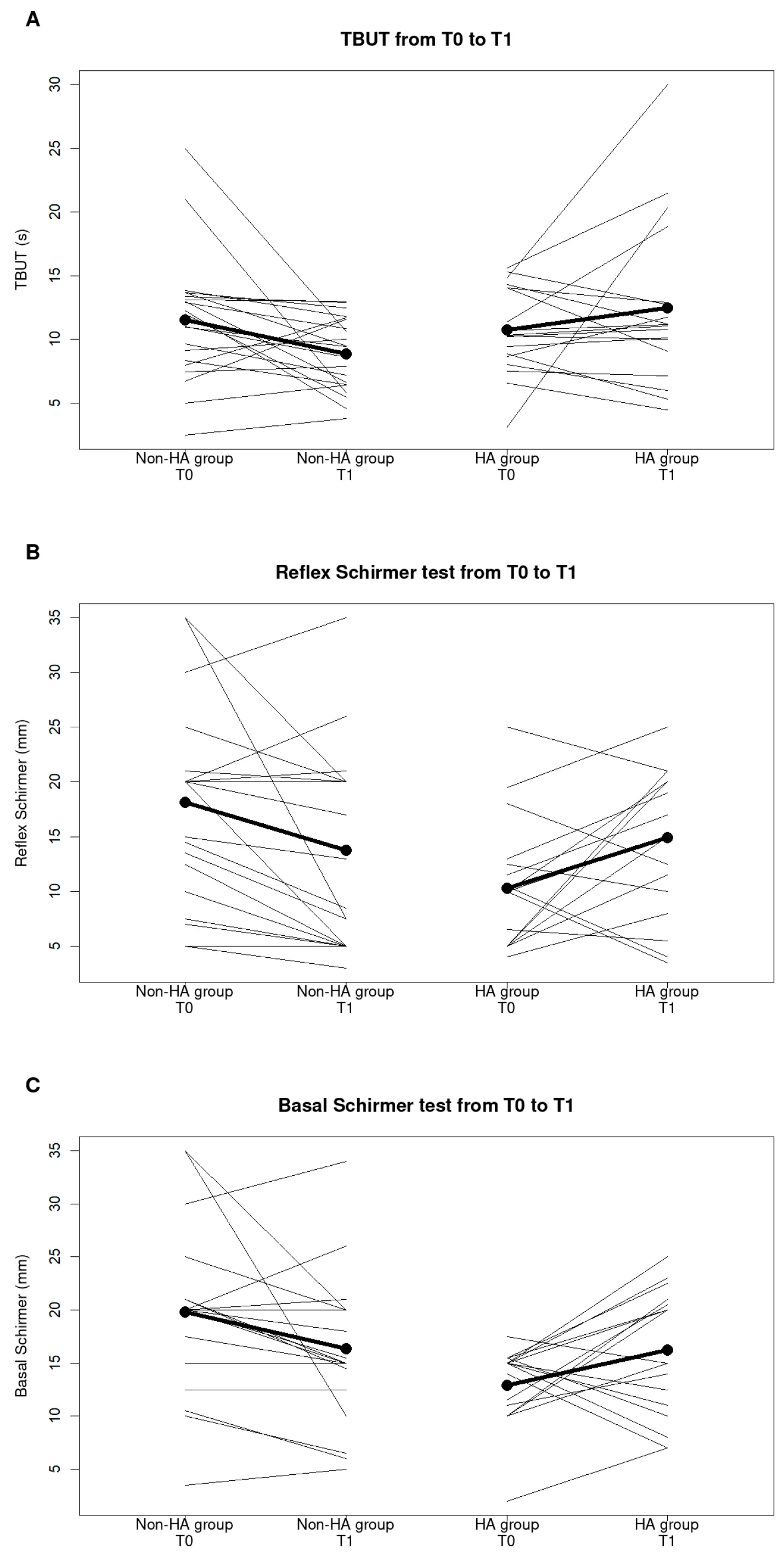
Individual ocular-surface changes from baseline (T0) to 4 months (T1) in the complete cohort according to HA use. (**A**) Tear break-up time (TBUT), (**B**) reflex Schirmer test, and (**C**) basal Schirmer test. Thin lines show within-patient changes, whereas thick black lines with filled circles represent group means. Abbreviations: HA, hyaluronic acid; TBUT, tear break-up time.

No adjusted between-group differences were observed for meibography (0.03; 95% CI, −0.18 to 0.24; *p* = 0.796; *q* = 0.796) or OSDI (−1.08 points; 95% CI, −3.51 to 1.35; *p* = 0.375; *q* = 0.469) (Table 2; Figure 2).

### 3.3. Low-Schirmer Subgroup

The low-Schirmer subgroup included 27 participants, of whom 18 were HA users and nine were non-HA users. Baseline objective ocular measurements were similar between groups, including TBUT (10.74 vs. 11.07 s), reflex Schirmer (10.31 vs. 10.00 mm), and basal Schirmer (12.92 vs. 13.89 mm). Baseline OSDI was higher in the HA group (11.03 vs. 1.92).

After adjustment for baseline values, TBUT at T1 was estimated to be 4.31 s higher in the HA group than in the non-HA group; however, the confidence interval included zero and the association was not significant after multiplicity correction (95% CI, −0.04 to 8.66; *p* = 0.052; *q* = 0.086).

**Figure 3:**
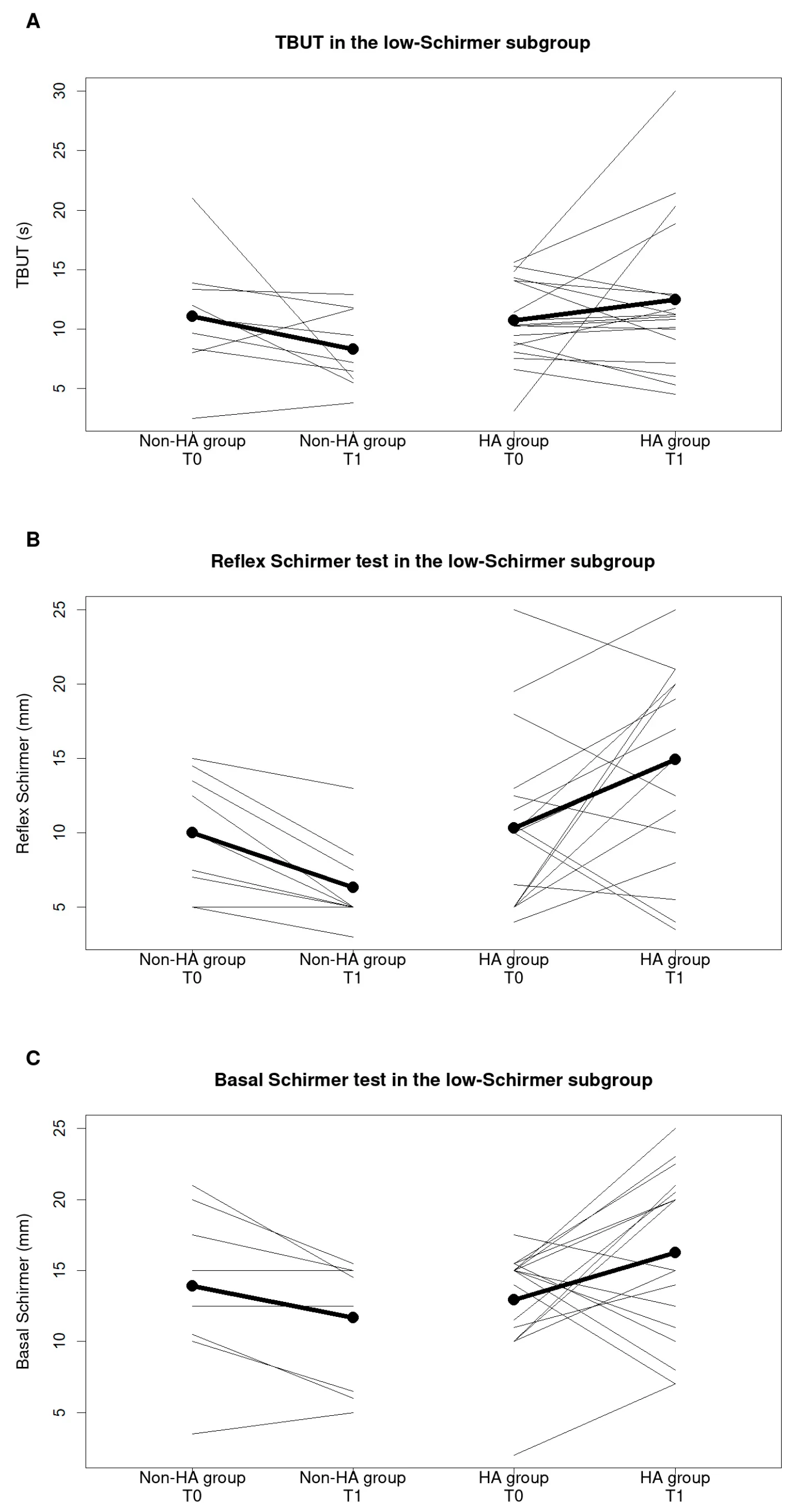
Individual ocular-surface changes from baseline (T0) to 4 months (T1) in the low-Schirmer subgroup according to HA use. (**A**) Tear break-up time (TBUT), (**B**) reflex Schirmer test, and (**C**) basal Schirmer test. Thin lines show within-patient changes, whereas thick black lines with filled circles represent group means. HA, hyaluronic acid; TBUT, tear break-up time.

However, reflex Schirmer at T1 was 8.50 mm higher in the HA group (95% CI, 4.84 to 12.17; *p* < 0.001; *q* < 0.001), and basal Schirmer was 5.07 mm higher (95% CI, 1.41 to 8.73; *p* = 0.009; *q* = 0.022). No clear between-group differences were observed for meibography or OSDI (Table 3; Figure 3).

### 3.4. Low-Schirmer Participants Without HA Eye Drops

Among the nine low-Schirmer participants of the non-HA group, mean reflex Schirmer decreased from 10.00 to 6.33 mm over 4 months. Eight of nine participants showed a reduction, corresponding to a mean change of −3.67 mm (paired Wilcoxon *p* = 0.014). TBUT decreased from 11.07 to 8.29 s in seven participants, although the within-group change was not statistically significant (mean change, −2.78 s; *p* = 0.129). Basal Schirmer decreased from 13.89 to 11.67 mm (mean change, −2.22 mm; *p* = 0.058).

**Figure 4:**
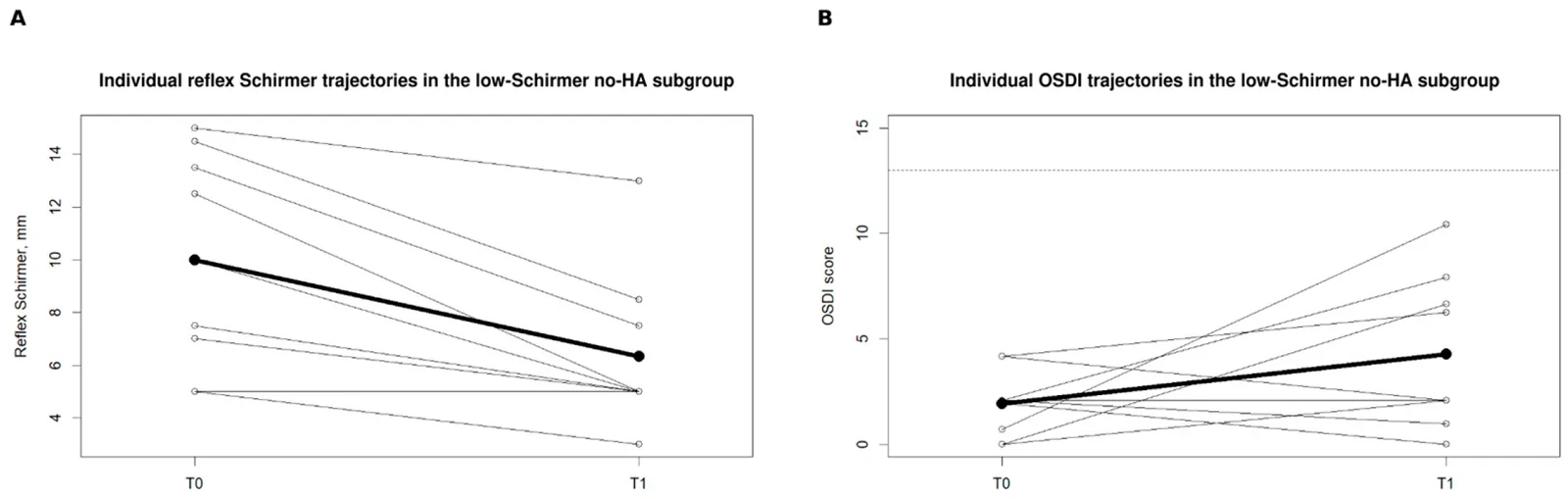
Individual ocular-surface changes from baseline (T0) to 4 months (T1) in the low-Schirmer non-HA subgroup. (**A**) Reflex Schirmer test and (**B**) Ocular Surface Disease Index (OSDI). Thin lines show within-patient changes, whereas thick black lines with filled circles represent group means. The dashed line indicates the conventional OSDI threshold of 13. Abbreviations: HA, hyaluronic acid; OSDI, Ocular Surface Disease Index.

Mean OSDI increased from 1.92 to 4.27 points (*p* = 0.183), but all nine participants had OSDI scores below 13 at both visits (Figure 4).

### 3.5. Eye-Level Sensitivity Analysis

The eye-level generalized estimating equation analysis confirmed the main findings obtained from the patient-level models. The estimated difference in T0-to-T1 change between groups was 4.38 s for TBUT (95% CI, 1.00 to 7.77; *p* = 0.011), 9.04 mm for reflex Schirmer (95% CI, 4.30 to 13.79; *p* < 0.001), and 6.76 mm for basal Schirmer (95% CI, 2.76 to 10.76; *p* = 0.001). No clear difference was observed for meibography (0.12; 95% CI, −0.24 to 0.47; *p* = 0.525).

### 3.6. Dermatologic Response

Dermatologic scores decreased markedly in both groups over the 4-month follow-up. Mean EASI decreased by 25.00 points in the non-HA group and by 24.89 points in the HA group. Mean SCORAD decreased by 53.10 and 62.30 points, respectively, whereas DLQI and pruritus NRS also improved substantially in both groups. After adjustment for the corresponding baseline values, no between-group differences were observed for EASI (adjusted difference, −1.79; 95% CI, −4.65 to 1.07; *p* = 0.212), SCORAD (−3.43; 95% CI, −11.82 to 4.95; *p* = 0.412), DLQI (0.43; 95% CI, −1.39 to 2.24; *p* = 0.637), or pruritus NRS (0.69; 95% CI, −1.14 to 2.51; *p* = 0.451). None of these comparisons was significant after false-discovery-rate correction. SCORAD sleep scores also decreased in both groups. In the non-HA group, the mean sleep score decreased from 6.7 ± 2.4 at T0 to 1.2 ± 1.4 at T1 (mean change, −5.5). In the HA group, it decreased from 7.6 ± 2.2 at T0 to 1.0 ± 1.2 at T1 (mean change, −6.6).

### 3.7. Association Between Dermatologic Severity and Ocular-Surface Parameters

At baseline, SCORAD was inversely correlated with reflex Schirmer (Spearman ρ = −0.483; *p* = 0.002; *q* = 0.009). No significant associations were observed between SCORAD and TBUT, basal Schirmer, meibography, or OSDI after false-discovery-rate correction. In the multivariable analysis adjusted for age, positive ophthalmologic history, and periocular eczema, each one-point increase in SCORAD was associated with a 0.25 mm lower reflex Schirmer value (adjusted coefficient, −0.250; 95% CI, −0.464 to −0.036; *p* = 0.023).

## 4. Discussion

In this prospective cohort, low Schirmer values, which can be regarded as subclinical markers of dry-eye disease, were very common, involving 69.2% of the recruited patients with AD. Interestingly, according to the literature [7,8,9], less than half of the subjects in the cohort reported ocular symptoms.

Ocular-surface parameters during the first 4 months of dupilumab treatment differed according to the use of HA eye drops. After baseline adjustment and correction for multiple comparisons, reflex Schirmer values were higher at follow-up in participants receiving HA. The findings were particularly notable in the low-Schirmer subgroup, in which subjects that were HA users and non-users reported similar baseline TBUT and reflex/basal Schirmer measurements. Within this subgroup, both reflex and basal Schirmer values were higher at follow-up among HA users.

The results are therefore consistent with an association between targeted early ocular-surface support (through the use of HA eye drops) and more favorable changes in tear production in patients with pre-existing objective impairment, but they should not be interpreted as evidence of treatment efficacy. This interpretation is clinically plausible given the evidence that ocular surface disease is frequently present before dupilumab initiation and that baseline dry-eye disease or impaired tear-film function may identify patients at increased risk of subsequent ocular adverse events [7,8,9,10,11,12].

An additional clinically relevant finding was the discrepancy between objective tear production and patient-reported symptoms. Among low-Schirmer participants who did not receive HA, reflex Schirmer decreased in eight of nine patients, while OSDI remained below the conventional abnormality threshold in all patients at both visits. Previous studies in AD have similarly shown that ocular-surface abnormalities may be present despite limited or absent symptoms [7,8,9]. This observation is also consistent with the broader dry-eye literature: in a recent analysis of 535 participants from the DRy Eye Assessment and Management (DREAM) study, discordance between symptoms and clinical signs was common [23]. Thus, symptom questionnaires alone may not adequately identify early changes in tear production during dupilumab treatment, particularly in patients with objective abnormalities at baseline. Nevertheless, OSDI and Schirmer testing assess different dimensions of ocular surface disease, and the clinical relevance of an isolated reduction in tear production in minimally symptomatic patients remains uncertain. These findings therefore support combining symptom assessment with objective ocular-surface evaluation in selected at-risk patients.

AD-related ocular disease and DAOSD have overlapping manifestations, including itching, conjunctival inflammation, blepharitis and tear-film disturbance [6,9]. Pre-existing or recurrent allergic/atopic inflammation may persist during treatment, while a new or worsening ocular disorder after dupilumab initiation raises the possibility of a treatment-associated component. Because of this clinical overlap, temporal association alone may be insufficient to establish causality.

The inverse association between baseline SCORAD and reflex Schirmer further suggests that ocular-surface vulnerability may partly reflect the underlying severity of AD rather than being exclusively treatment-induced. Greater baseline AD severity has previously been associated with a higher risk of conjunctivitis during dupilumab treatment [5], and ocular surface disease is highly prevalent before treatment initiation [8,9]. Baseline sleep scores were somewhat higher in the HA group than in the non-HA group (7.6 vs. 6.7), suggesting that sleep disturbance may have contributed in part to the higher baseline SCORAD observed in this group. Mechanistic observations also support an interaction between pre-existing susceptibility and treatment-related changes in ocular-surface homeostasis: conjunctival goblet-cell dysfunction has been described during dupilumab treatment [24], while recent transcriptomic data suggest that patients who subsequently develop ocular adverse events may already display a distinct conjunctival molecular profile before dupilumab exposure [25]. Our findings also extend the limited literature on preventive ocular management during dupilumab treatment. Previous studies have mainly evaluated clinically diagnosed conjunctivitis: individualized ophthalmologic management including artificial tears was associated with a lower observed incidence of conjunctivitis in a nonrandomized study [15], and prophylactic diquafosol was associated with a lower incidence in an open-label prospective study [16]. Current expert consensus, however, considers the evidence insufficient to recommend routine prophylactic lubricant eye-drop use in patients without pre-existing ocular disease [13]. Our study addresses a different question because HA eye drops were prescribed selectively rather than universally. Given the established use and favorable safety profile of HA-containing artificial tears in dry-eye disease [14], our observations support a risk-stratified approach, even if it does not establish that HA prevents DAOSD. Before dupilumab initiation, ocular history and current symptoms should be reviewed; ophthalmology assessment is particularly relevant for significant pre-existing or active disease. Persistent symptoms, pain, photophobia or visual change warrant appropriate specialist assessment. Routine comprehensive ophthalmologic testing for every asymptomatic patient is not established by this small cohort [13].

Ocular events have also been reported in other dermatologic populations treated with dupilumab. Conjunctivitis was reported in the PRIME/PRIME2 trials in prurigo nodularis [26] and in a multicenter cohort of patients with bullous pemphigoid treated with dupilumab [27], whereas in chronic spontaneous urticaria conjunctivitis was not increased with dupilumab compared with placebo in the LIBERTY-CSU CUPID trials [28]. Thus, ocular events are not exclusive to AD, although their occurrence may vary across indications.

Management should follow the ocular phenotype and severity. Alternatives or additions to HA include other preservative-free lubricants and lid care, with antihistamine/mast-cell-stabilizing drops when allergy contributes. Persistent inflammation may require ophthalmologist-supervised topical corticosteroids, ciclosporin or selected tacrolimus treatment [13,29]. Diquafosol remains supported by limited preventive evidence [16]. Uncontrolled ocular disease may require joint dermatologic–ophthalmologic consideration of systemic treatment modification. This cohort provides no comparative evidence for choosing among these approaches.

This study has several strengths, including prospective ophthalmologic assessment before and after dupilumab initiation, combined evaluation of objective and patient-reported ocular outcomes, baseline-adjusted analyses with correction for multiple comparisons, and sensitivity analyses accounting for within-patient correlation between eyes. However, important limitations must be acknowledged. Lubricant allocation was not randomized and was determined by baseline ocular findings and symptoms, creating confounding by indication; although baseline adjustment and restriction to the low-Schirmer subgroup reduced some of the imbalance, residual confounding cannot be excluded. Several baseline demographic and dermatologic characteristics were similar between groups; however, differences in SCORAD, allergic conjunctivitis, allergic rhinitis, and some previous systemic treatments may also have contributed to residual confounding. Previous ocular treatments and concomitant medications during follow-up were not systematically recorded and could not be reliably compared between groups. Demodex infestation, which may contribute to blepharitis and ocular-surface symptoms [30], was not systematically assessed in this cohort and therefore represents a potential unmeasured confounder. The sample size was limited, particularly among untreated low-Schirmer participants, and the single-center design and 4-month follow-up restrict precision and generalizability. Baseline overlap was also limited for basal Schirmer in the complete cohort, making that estimate less robust. Accordingly, the observed differences should be interpreted as associations. Overall, the findings suggest that baseline ophthalmologic assessment may identify patients with reduced tear production that is not fully reflected by symptoms and who may warrant closer objective monitoring during dupilumab treatment. Larger multicenter studies with controlled treatment allocation and longer follow-up are needed to determine whether targeted early ocular-surface support can prevent clinically relevant DAOSD.

## 5. Conclusions

In this prospective cohort, HA supplementation at dupilumab initiation was associated with more favorable changes in tear production, particularly among patients with reduced baseline Schirmer measurements, while the nonrandomized design of the study does not consent to establish a protective effect of HA. Objective deterioration in tear production was observed in several untreated low-Schirmer patients despite persistently low OSDI scores, highlighting the potential limitation of symptom-based assessment alone. These findings highlight the potential value of baseline ophthalmologic evaluation and combined objective and symptom monitoring during dupilumab treatment. Larger controlled studies are needed to determine whether targeted early HA eye-drop use can prevent clinically relevant DAOSD.

## Figures and Tables

**Table 1:** Baseline demographic, dermatologic, and ocular characteristics in non-HA and HA groups.

Characteristic	Non-HA Group (*n* = 21)	HA Group (*n* = 18)	*p* Value	SMD
**Demographic and dermatologic characteristics**				
Age at dupilumab initiation, years	40.81 ± 19.85	41.11 ± 20.29	0.963	0.02
Female sex	11/21 (52.4%)	10/18 (55.6%)	1.000	0.06
EASI, score	29.28 ± 7.06	27.56 ± 4.07	0.350	−0.30
SCORAD, score	65.03 ± 14.90	73.63 ± 14.43	0.076	0.59
DLQI, score	12.38 ± 6.24	14.94 ± 6.50	0.219	0.40
Pruritus NRS, score	8.43 ± 1.86	8.78 ± 2.21	0.600	0.17
**Ocular clinical features**				
Positive ophthalmologic history	11/21 (52.4%)	11/18 (61.1%)	0.748	0.18
History of allergic conjunctivitis	9/21 (42.9%)	12/18 (66.7%)	0.201	0.49
Periocular eczema	4/21 (19.0%)	3/18 (16.7%)	1.000	−0.06
**Ocular surface characteristics**				
Tear break-up time, seconds	11.55 ± 4.96	10.74 ± 3.43	0.552	−0.19
Reflex Schirmer, mm	18.14 ± 8.82	10.31 ± 5.78	0.002	−1.05
Basal Schirmer, mm	19.81 ± 7.67	12.92 ± 3.66	<0.001	−1.15
Meibography, mean grade	1.57 ± 0.60	1.33 ± 0.49	0.178	−0.44
OSDI, score	2.31 ± 2.60	11.03 ± 14.07	0.018	0.86

Data are presented as mean ± standard deviation or n/N (%), as appropriate. *p* Values are descriptive and were calculated using Welch’s *t* test for continuous variables and Fisher’s exact test for categorical variables. SMDs were calculated as the HA group minus the non-HA group and were applied to both continuous and binary variables; for binary variables, such as female sex, the SMD represents the standardized difference between group proportions. Bilateral ocular measurements represent the mean of the right and left eyes. Abbreviations: DLQI, Dermatology Life Quality Index; EASI, Eczema Area and Severity Index; NRS, numerical rating scale; OSDI, Ocular Surface Disease Index; SCORAD, SCORing Atopic Dermatitis; SMD, standardized mean difference.

**Table 2:** Ocular outcomes at 4 months according to HA eye-drop use.

Outcome	Mean Change Non-HA Group	Mean Change HA Group	Adjusted T1 Difference (95% CI)	*p* Value	*q* Value
Tear break-up time, seconds	−2.65	1.73	3.81 (0.39 to 7.24)	0.030	0.076
Reflex Schirmer, mm	−4.40	4.64	5.60 (1.64 to 9.57)	0.007	0.035
Basal Schirmer, mm	−3.43	3.33	3.12 (−1.00 to 7.24)	0.133	0.222
Meibography, mean grade	−0.31	−0.19	0.03 (−0.18 to 0.24)	0.796	0.796
OSDI, score	1.02	−1.96	−1.08 (−3.50 to 1.35)	0.375	0.469

Changes are mean T1 minus T0 values. Adjusted differences represent HA group minus no-HA group at T1 and were estimated by analysis of covariance adjusted for the corresponding T0 value. Abbreviations: CI, confidence interval; OSDI, Ocular Surface Disease Index.

**Table 3:** Baseline-adjusted ocular outcomes at 4 months in the low-Schirmer subgroup.

Outcome	Baseline Mean No-HA Group (*n* = 9)	Baseline Mean HA Group (*n* = 18)	Adjusted T1 Difference (95% CI)	*p* Value	*q* Value
Tear break-up time, s	11.07	10.74	4.31 (−0.04 to 8.66)	0.052	0.086
Reflex Schirmer, mm	10.00	10.31	8.50 (4.83 to 12.17)	<0.001	<0.001
Basal Schirmer, mm	13.89	12.92	5.07 (1.41 to 8.73)	0.009	0.022
Meibography, mean grade	1.83	1.33	0.17 (−0.11 to 0.45)	0.224	0.224
OSDI, score	1.92	11.03	−2.40 (−6.09 to 1.29)	0.193	0.224

The low-Schirmer subgroup comprised participants with at least one baseline basal or reflex Schirmer measurement ≤15 mm in either eye. Adjusted differences represent HA group minus no-HA group at T1 and were estimated using linear analysis of covariance adjusted for the corresponding baseline value. *q* values were calculated using the Benjamini–Hochberg false-discovery-rate procedure across the five ocular outcomes. For TBUT and Schirmer measurements, positive adjusted differences indicate higher T1 values in the HA group. For meibography and OSDI, lower values indicate a more favorable outcome. Abbreviations: CI, confidence interval; OSDI, Ocular Surface Disease Index.

## Data Availability

The original contributions presented in this study are included in the article. Further inquiries can be directed to the corresponding author.

## Abbreviations

The following abbreviations are used in this manuscript:

AD	atopic dermatitis
BUT	break-up time
CI	confidence interval
DAOSD	dupilumab-associated ocular surface disease
DLQI	Dermatology Life Quality Index
DREAM	Dry Eye Assessment and Management
EASI	Eczema Area and Severity Index
HA	hyaluronic acid
HC3	heteroskedasticity-consistent type 3
IL	interleukin
NRS	numerical rating scale
OSDI	Ocular Surface Disease Index
P2Y2	purinergic receptor P2Y2
SCORAD	SCORing Atopic Dermatitis
SMD	standardized mean difference
T0	baseline
T1	4-month follow-up
TBUT	tear break-up time
